# Classical and Unexpected Effects of Ultra-Micronized PEA in Neuromuscular Function

**DOI:** 10.3390/biom12060758

**Published:** 2022-05-29

**Authors:** Pierangelo Cifelli, Gabriele Ruffolo, Marco Ceccanti, Chiara Cambieri, Laura Libonati, Eleonora Palma, Maurizio Inghilleri

**Affiliations:** 1 Department of Applied Clinical and Biotechnological Sciences, University of L’Aquila, 67100 L’Aquila, Italy; 2 Department of Physiology and Pharmacology, Istituto Pasteur-Fondazione Cenci Bolognetti, University of Rome Sapienza, 00185 Rome, Italy; gabriele.ruffolo@uniroma1.it (G.R.); eleonora.palma@uniroma1.it (E.P.); 3 IRCCS San Raffaele Roma, 00163 Rome, Italy; 4 Department of Human Neuroscience, University of Rome Sapienza, 00185 Rome, Italy; marco.ceccanti@uniroma1.it (M.C.); chiara.cambieri@uniroma1.it (C.C.); laura.libonati@uniroma1.it (L.L.)

**Keywords:** palmitoyl ethanolamide, neuromuscular junction, neurophysiology

## Abstract

Recently, the endocannabinoid system has attracted growing attention from the scientific community for its involvement in homeostatic and pathological processes as they pertains to human physiology. Among the constituents of the endocannabinoid system, the molecule palmitoyl ethanolamide has particularly been studied for its ability to reduce several inflammatory processes involving the central nervous system. Here, we reviewed published literature and summarized the main targets of the palmitoyl ethanolamide, along with its unique possible mechanisms for restoring correct functioning of the central nervous system. Moreover, we have highlighted a less-known characteristic of palmitoyl ethanolamide, namely its ability to modulate the function of the neuromuscular junction by binding to acetylcholine receptors in different experimental conditions. Indeed, there are several studies that have highlighted how ultra-micronized palmitoyl ethanolamide is an interesting nutraceutical support for the treatment of pathological neuromuscular conditions, specifically when the normal activity of the acetylcholine receptor is altered. Although further multicentric clinical trials are needed to confirm the efficacy of ultra-micronized palmitoyl ethanolamide in improving symptoms of neuromuscular diseases, all the literature reviewed here strongly supports the ability of this endocannabinoid-like molecule to modulate the acetylcholine receptors thus resulting as a valid support for the treatment of human neuromuscular diseases.

## 1. Introduction

Palmitoyl ethanolamide [PEA] represents the best-known endocannabinoid-like molecule of the “autacoid local injury antagonist amide” [ALIAmide] family. ALIAmides are endogenous N-acyl ethanol amines [NAEs] with homeostatic functions able to modulate different physiological pathways involved in inflammation, pain, and metabolism [1]. Among the different ALIAmides, discovered up to now, PEA, oleoyl ethanol amide (OEA), stearoyl ethanol amide (SEA), and anandamide (ANA) represent the most-studied compounds, while others such as eicosatrienoyl ethanol amide (ETEA) still need to be investigated further. In this review we will highlight the different mechanisms of action of PEA, an endocannabinoid [eCB]-like bioactive lipid mediator, and its involvement in different human pathologies. Finally, we will place under the spotlight PEA’s ability to modulate the synaptic function in the neuromuscular system and at neuromuscular junction [NMJ].

## 2. What, Where and Why?

NAEs are endogenous bioactive lipids characterized by common precursors, namely the N-acylated ethanolaminephospholipids such as N-acylphosphatidylethanolamines (NAPEs) and N-acylplasmenylethanolamines (pNAPEs). The biological targets and the specific functions of the different NAEs depend upon the origin of the N-acyl group, which can arise from a variety of different fatty acids [2]. The endogenous levels of NAEs in different tissues are mainly regulated by enzymatic reactions responsible for both their biosynthesis and degradation. Two main enzyme reactions are involved in NAEs biosynthesis. The first is the process of N-acylation of ethanolamine phospholipids mediated by a Ca^2+^-dependent N-acyltransferase, while the second is represented by the release of the different NAEs from NAPEs, mediated by NAPE-hydrolyzing phospholipase (NAPE-PLD) [3]. Catabolism of NAEs is rapidly put in effect by the action of the free acid amide hydroxylase [FAAH], an integral membrane enzyme able to hydrolyze them in their corresponding fatty acids and ethanolamine [4].

Among NAEs, PEA was first identified in mammalian tissues in 1965 [5]. In central nervous system [CNS] it is produced and released both from neurons and glial cells as an “on-demand molecule” to promote endogenous neuroprotection following tissue damage and the subsequent processes of neuroinflammation. PEA is part of the endocannabinoid system (ECS), a regulatory signaling network made up of lipidic molecules, the endocannabinoids (eCBs), their receptors, and different enzymes involved in the metabolism and catabolism of the lipidic signals. The ECS is strongly involved in different homeostatic processes of our body, both in physiological and pathological conditions [6]. Indeed, the ECS is implicated in the regulation of several functions, such as the cycle of sleep, the regulation of food intake, and the perception of painful stimuli and in high cognitive functions such as mood regulation, memory, and reward [7].

As mentioned, PEA can carry out a plethora of actions, and its tissue levels are often up regulated in several pathological conditions [8]. Its capacity to modulate divergent pathways is largely due to its ability to interact, directly or indirectly, with different targets [9]. The main target of PEA is represented by the nuclear peroxisome proliferator-activated receptor-α (PPAR-α) [10]. This receptor, part of the larger family of nuclear receptor proteins, has the capability, when activated, to modulate and regulate the expression of different genes involved in inflammation [11]. Indeed, the activation of PPAR-α receptors induces and promotes a cascade of events leading to inhibition and suppression of the release of pro-inflammatory cytokines, such as Interleukin-1β (IL-1β), Interleukin 6 (IL6), and the tumor-necrosis factor (TNF-α) [12]. All these actions were further confirmed in several pre-clinical studies, where the anti-inflammatory effect of PEA was totally abolished when applied on PPAR-α knockout models of disease [12]. Another target activated by PEA is represented by GPR55 and GPR119, two orphan receptors [13], considered up to now as novel cannabinoid receptors [14]. PEA is also able to activate and modulate the transient receptor potential vanilloid receptor 1 (TRPV1) channels, accounting thus, at least in part, for its anti-nociceptive effect [15]. Furthermore, PEA can indirectly activate the canonical cannabinoid receptors, namely CB1 and CB2, increasing the level of anandamide (AEA) by inhibiting its enzymatic-mediated hydrolysis carried out by fatty acid amide hydrolase (FAAH) [16].

One of the main targets of PEA is represented by the resident immune cells of CNS, namely microglial cells. These latter are responsible for CNS homeostasis, being able to modify their function following CNS injuries [17]. Indeed, after multiple insults, [e.g., inflammation, tumors, and traumas], they can switch their phenotype between the M1, considered unanimously the pro-inflammatory, and the M2, considered instead as anti-inflammatory [18]. The imbalance between the two states is considered as one of the main events underlying many neurogenerative diseases, where M1 phenotypes are found in a condition of continuous activation [19]. In this state, M1-activated microglia releases many inflammatory cytokines, such as t TNF-α, IL-1β, and inducible reactive oxygen species (iROS), which lead the neuronal population to death [19]. Recent studies demonstrated that PEA can modulate microglia polarization, reducing the release of pro-inflammatory cytokines and promoting migration and phagocytic activity [20]. In detail, PEA can reduce the lipopolysaccharide (LPS)-induced microglial activation by switching their phenotypic constructs from M1 to M2, thus inhibiting the release of pro-inflammatory elements in the extracellular milieu and reducing the hyperexcitability of cultured primary cortical cells together with LPS-activated microglial cells [21]. Notably, these effects were all mediated by cannabinoid receptor type 2 (CB2), thus confirming the strong involvement of the ECS during inflammatory processes [22].

## 3. Pharmacology of PEA

PEA is a lipophilic molecule with low solubility rate. Hence, specific formulations, such as micronized and ultra-micronized PEA, have been used to increase its solubility and enhance its bioavailability. Indeed, with these techniques PEA particles became smaller, thus increasing surface areas to improve absorption [23]. Different studies highlight that ultra-micronized PEA has greater absorbability than micronized and non-micronized PEA, also showing better results in terms of reducing pain perception [24,25,26] Another issue about PEA is that its bioavailability is related to its possible pre-systemic metabolism. Since enzymes involved in its metabolism are expressed both in the small intestine and in the liver [27], it is quite difficult to calculate its range of distribution and how this parameter may change in disparate individuals.

Due to its partially unclear mechanisms of absorption and pre-systemic metabolism, the distribution mechanism of PEA is still far from being elucidated. However, due to its lipophilic nature, several studies demonstrated that, even with its low level of bioavailability, PEA its able to reach tissues to target its receptors effectively [28,29]. Currently, the only way to characterise its absorption is by measuring its blood levels after administration [28].

Once PEA reaches the tissues, there is a fast cellular uptake in order to expose PEA to its intracellular PPAR-α receptors. This crucial step needs to be further deciphered, as research pertaining to its mechanism is still lacking. It appears that the intracellular process of uptake is strictly regulated by PEA hydrolysis, to balance its extracellular/intracellular ratio [30]. In contrast with data about absorption and availability, studies about PEA metabolism are abundant in literature. Indeed, PEA is hydrolysed in two compounds, namely palmitic acid and ethanolamine by the fatty acid amide hydrolase [FAAH] enzyme, located on endoplasmic reticulum membranes [31,32]. Subsequently, another enzyme involved in PEA hydrolysis was found and described: the FAAH-2 [33]. Notably, both FAAH and FAAH-2 are able to hydrolase AEA faster than PEA, while the third enzyme involved in PEA catabolism, namely N-acylethanolamine acid amidase (NAAA), is able to hydrolase more efficiently PEA than AEA [34,35].

The reason why three distinct enzymes are involved in PEA catabolism is still obscure. Nevertheless, recent works have highlighted the possibility that it may depend on the source of PEA, with differences between endogenous and exogenous PEA. Endogenous PEA is hydrolysed by means of both FAAH and NAAA, but several studies indicate that the prevalence of one or the other is strongly tissue-specific and may change also in different pathological conditions [36]. 

Alterations were described when catabolism of exogenous PEA was scrutinized; indeed, the fatty acid binding proteins do not deliver exogenous PEA to lysosomal NAAA, thus suggesting a prevalent role of FAAH for exogenous PEA metabolism [37].

The catabolism of the main metabolite of PEA, palmitic acid, was fully described by Carta et al. in 2017 [38]. However, data describing the excretion of unmetabolized PEA is still lacking, even though the kidney route seems to be prominent [5].

## 4. PEA, a Multi Target Drug for Different Clinical Applications

Since, as mentioned above, PEA can bind different receptor targets and is thus able to modulate different signalling pathways, its rationale for clinical uses embraces a wide variety of pathological conditions, from chronic inflammations to perseverant pain conditions. Here we will address the main applications in routinely clinical practice with a particular focus on neurological conditions.

## 5. PEA and Neuroinflammation

Nowadays, neuroinflammation is considered a key element in the pathogenesis of a broad spectrum of neurological diseases, from neurodegenerative conditions, such as Alzheimer’s disease [39,40,41,42,43] and Parkinson’s disease [44], to traumatic diseases, such as traumatic brain injuries [TBI] [45,46,47,48,49], strokes [50], and other conditions where neuronal excitability is increased, such as epilepsy [51]. Moreover, recently, attention has been called to the significant effect of PEA in clinical conditions involving the higher functions of CNS, such as cognitive impairment [52] and mood disorders [53]. In all these conditions, microglial cells, together with mast cells and astrocytes, play a crucial role in inducing and maintaining the inflammatory processes, leading to neuronal cell death, and thus impairing the CNS functions. PEA capacity to dampen neuroinflammation could represent a viable tool to preserve neuronal populations and to retain physiological brain functions [54,55]. Most of PEA’s effects are due, as already mentioned, to its ability to modulate and modify microglial, astrocyte and mast cells activation [56,57]. Indeed, PEA can enhance microglial migration without switching their phenotype towards the pro-inflammatory M1 state, thus increasing the resistance to infections without activation of inflammatory cascade [57]. PEA is able to carry out its neuroprotective effects on CNS also with other mechanisms, like for example, inhibiting the apoptosis processes and modulating different pathways, such as the bax/bcl-2 and Akt/mTOR/p70S6K pathways [58]. PEA is also able to target and modulate NMDA receptors, thus protecting cells for glutamate toxicity [59]. PEA neuroprotective actions are also carried out by promoting and modulating synaptic homeostasis and favouring neurogenesis [60,61,62]. Notably, all these PEA-mediated actions were confirmed using different animal models of disease characterized by neuroinflammation. Chronic administration of PEA at different concentrations (ranging from 10 to 100 mg/Kg/day) was able to significantly reduce neuroinflammation [63,64,65], to protect neurons from death [21,66], to reduce iROS productions [66], and to improve behavioural, motor and cognitive deficits [67,68,69].

## 6. PEA in Central Nervous System Diseases

It is well known that both ECS and NAEs are strongly involved in regulation and modulation of behaviour, cognition and in mood regulation [70]. Plasmatic PEA levels are significantly altered in conditions as post-traumatic stress disease (PTSD) [71], depression [72,73], and in autism spectrum disorders (ASD) [74]. The mechanisms by which PEA can improve these conditions are different and include increased hippocampal neurogenesis [59,60,61] and increased maturation of oligodendrocyte precursor cells [59]. PEA was able to prevent the plasmatic reduction of the brain-derived neurotrophic factor (BDNF) and glial cell line-derived neurotrophic factor (GDNF) in a murine model of stroke [75], thus explaining, at least in part, its neuroprotective effects. Interestingly, PEA is also strongly involved in the neuromodulation of GABAergic transmission. As a matter of fact, PEA by binding GPR55 receptors is able to enhance GABAergic transmission in the *corpus striatum* and to increase the synthesis of the endocannabinoid 2-AG at postsynaptic level thus modulating GABA release through the stimulation of presynaptic CB1Rs [76]. These biological effects may partially explain the neuroprotective action of PEA and its potential use to treat these patients in clinical activities. To confirm the pre-clinical results obtained by using PEA, human studies were performed on different pathological diseases. In a yearlong study, PD patients used ultra-micronized PEA as add-on therapy along with levodopa, showing a significant improvement on both motor- and non-motor-symptoms, ameliorating the typical mood deficits of Parkinsonian patients as well as reducing fatigue and improving sleep-cycle coupled with improved responses to different mental tasks [77]. Notably, in a double-blind, randomized, placebo-controlled study performed on patients with major depressive disorder [MDD], 600 mg of ultra-micronized PEA used as add-on therapy with typical selective serotonin reuptake inhibitors [SSRI] for 6 weeks was able to significantly improve the depressive scores, ameliorating symptoms when compared to SSRI plus placebo group [78]. It is important to note that, also if further clinical studies are required in all the human studies performed to date, PEA was well tolerated and displayed only few and minor adverse effects [79].

## 7. Analgesic Properties of PEA

The direct link between inflammation and pain kicked off a series of studies with the aim to emphasize PEA analgesic properties. Taking advantage of pre-clinical models of inflammation and neuropathic pain, it was possible to demonstrate the strong analgesic properties of PEA [80,81,82]. When inflammation processes persist, PEA levels increase as a protective mechanism to inhibit inflammatory pathways leading to tissue damage and loss of functions. If the stimulus is protracted, PEA levels start to decrease, thus indicating that PEA’s increased concentration is not enough to restore the physiological conditions when inflammation becomes chronic [83]. The PEA’s analgesic properties are guaranteed by several mechanisms. For instance, its ability to directly bind to PPAR-α and GPR55 receptors or to indirectly bind to CB1, CB2, and TRPV1 receptors may partially account for its properties [15]. The analgesic properties of PEA also take into account its capacity to suppress inflammation by reducing mast cells activation and to reduce the production and the release of different inflammatory mediators such as nerve growth factor (NGF), cyclo-oxygenase-2 (COX-2), TNF-α, and iROS [84]. All these pathways are responsible for the preservation of peripheral nerve morphology and for reducing inflammation-coupled oedema and microglial activation [85,86]. In addition, several preclinical and clinical studies confirm the anti-nociceptive properties of PEA in neuropathic pain [87,88]. PEA treatment was also able to preserve Langerhans islets’ morphology in the pancreatic gland of diabetic mice [89]. Orefice et al., in a randomized, double-blind, placebo-controlled study, demonstrated that ultra-micronized PEA as add-on therapy in patients afflicted by multiple sclerosis was able to significantly reduce the pain sensation and to improve their quality of life [90].

Notably, currently available drugs used to treat inflammation and pain-related conditions often display several adverse effects, especially when used chronically, such gastrointestinal, hepatic, renal, and cardiovascular disorders. PEA thus represents a promising alternative treatment, since it shows a high profile of safety and tolerability [78].

## 8. PEA in Neuromuscular Alterations and Diseases

The first study that hypothesized a potential use of ultra-micronized PEA in pathological conditions involving neuromuscular transmission was published in 2012 by Clemente who described the improvement of different vital parameters in a single case of a patient affected by amyotrophic lateral sclerosis (ALS), a neurodegenerative disorder able to disrupt the physiological function of the neuromuscular junction leading, in a few years, to muscle paralysis and death of the patients [85]. Up to now, there are several studies that highlight some of the possible mechanisms involved in this pathology, but unfortunately, ALS is still a disease with a poor prognosis lacking effective therapies and treatments in spite of recent efforts to develop new therapeutic strategies [91,92]. The degeneration of the second motor neuron is considered a hallmark of this disease, but different studies performed on animal models of ALS highlighted that the first alterations are localized at the neuromuscular junction (NMJ) [93,94]. Indeed, it was described that, even during the pre-symptomatic period, changes in NMJ function and muscular functions are present, thus leading to the “dying-back” theory [95]. According to this hypothesis, the first pathogenic step in ALS is represented by strong functional alterations at the NMJ level leading subsequently to the progression of the disease involving motor neuron degeneration [96], muscle atrophy, mitochondrial dysfunction, microgliosis, and neuronal degeneration [97]. To strengthen this hypothesis, it was demonstrated that muscular acetylcholine receptors (AChRs) obtained from muscle biopsy of ALS patients are functionally impaired when compared with AChRs obtained from non-ALS denervated muscle, showing also a reduction of sensitivity to ACh 98]. In detail, it is well known that after denervation, muscular AChRs subunits’ composition undergoes modification, with a wide up-regulation of the α1β1γδ (γ-AChR) on the muscular sarcolemma. Indeed, in ALS muscles where both denervation and aberrant reinnervation are present, both the γ-AChR and the ε-AChRs are expressed. It was demonstrated that PEA can significantly reduce the rate of AChR desensitization of receptors obtained from ALS patients, during repetitive application of ACh [98]. In detail PEA was able to reduce this functional property predominantly on the ε-AChRs form, while this effect was to a lesser extent on γ-AChR [98]. To further strengthen this observation in the same study, an observational double-blind clinical test was performed on a cohort of ALS patients recruited by the neurological unit at the Policlinico Umberto I in Rome headed by Inghilleri. In detail, 28 patients were co-treated with riluzole (at the time of the study, the only officially approved treatment) [99] plus ultra-micronized PEA twice a day (50 mg and 600 mg, respectively) and compared with 36 patients receiving only riluzole. The two groups of patients were monitored, and clinical and electrophysiological test were assessed. Interestingly, ultra-micronized PEA-treated patients showed a slow respiratory impairment and a delayed need of tracheotomy. This clinical improvement was associated with a reduced decay of the forced vital capacity (FVC) in comparison with patients treated only with riluzole [100]. Therefore, the authors suggested that the PEA’s effect on FVC is due to its ability to reduce the muscle AChRs desensitization induced by repetitive stimulation, as in respiratory muscles. Taken together, data obtained from this study showed that PEA add-on therapy slows the disease progression, suggesting that PEA could represent a valid aid to slow respiratory impairment in these patients, thus increasing their life expectancy. In another open-label pilot study, also carried out by Inghilleri’s group at the Policlinico Umberto I in Rome, oral ultra-micronized PEA was administered to a cohort of patients afflicted by myasthenia gravis (MG) [101]. This autoimmune disease is characterized by the presence of auto-antibodies able to disrupt the physiological function of NMJ. Symptoms are often variable, from mild deficits limited to single muscle groups to generalized weakness involving vital functions. However, the main symptom is muscular fatigue, which causes characteristic fluctuations in symptoms that worsen in the evening and improve after periods of rest. The progression of the disease can be sneaky, with long periods of spontaneous remission or rapid progression [102]. In the aforementioned study [101], after only one week of treatment, patients displayed a significant improvement of all the neurophysiological parameters taken in account. In detail, ultra-micronized PEA induced a statistically significant improvement of the quantitative myasthenia gravis score (QMG), thus reducing the level of disability of the treated patients, and also a significant effect in improving the pathological muscle responses (measured as “decremental muscle responses”, RNS). Since these positive effects appeared after only one week of treatment, it is not surprising that they disappeared one week after the withdrawal of the ultra-micronized PEA. Notably, the antibody titre did not significantly change following PEA treatment [101], suggesting a possible direct effect of ultra-micronized PEA on nAChRs as already shown in ALS patients. The capacity of PEA to reduce the release of pro-inflammatory cytokines could also be exploited to treat diseases such as sarcopenia, a condition characterized by progressive and generalized loss of skeletal muscle mass and strength combined with low physical performance [103]. Recently, new efforts have been made to improve PEA bioavailability, in order to better reach peripheral cells such as muscle cells [104], opening thus new scenarios in the treatments of diseases characterized by strong inflammation. On the other hand, new insights on the use of ultra-micronized PEA on inflammatory pathways modulation came from studies using PEA in association with antioxidant agents, such as phycocyanin extract (PC) from spirulina algae [66], that further enhanced PEA’s beneficial properties. Indeed, a multi-center, double-blinded, randomized placebo-controlled clinical trial showed that co-administration of PEA plus Luteolin was able to significantly improve the recovery of olfactory function in patients after SARS-CoV-2 infection [105]. 

## 9. Conclusions

PEA signifies a valid support in different pathological conditions, since it displays a wide range of positive effects without noteworthy side effects. Until now, PEA has been extensively used to treat and ameliorate conditions characterized by neuroinflammation and to treat and reduce the perception of neuropathic pain. It is possible to find separate commercially available PEA formulations, (micronized, ultra-micronized), which increase its solubility and bioavailability, and in doses ranging from 200 mg to up to 1000 mg. While its neuroprotective effect is well known, its capacity to interact with the NMJs deserves further consideration, as it could pave the way for novel therapeutic implications. In detail, the ability to modulate the AChR function could open up new perspectives for its use in different pathological conditions characterized by alterations of NMJ, such as ALS, but also in other diseases such as MG where AChRs function is strongly altered. Further studies including larger double-blind multicentre clinical studies are required in order to find the best medical approach for any condition taken into consideration.
